# Synthesis and Properties of Low-Molecular-Weight PEI-Based Lipopolymers for Delivery of DNA

**DOI:** 10.3390/polym10101060

**Published:** 2018-09-25

**Authors:** Miao-Miao Xun, Zheng Huang, Ya-Ping Xiao, Yan-Hong Liu, Ji Zhang, Ju-Hui Zhang, Xiao-Qi Yu

**Affiliations:** 1 National Demonstration Center for Experimental Chemical Engineering Comprehensive Education, School of Chemical Engineering and Technology, North University of China, Taiyuan 030000, China; 2 Key Laboratory of Green Chemistry and Technology (Ministry of Education), College of Chemistry, Sichuan University, Chengdu 610064, China; huangzheng1026@126.com (Z.H.); 15982103680@163.com (Y.-P.X.); yanhongliu@scu.edu.cn (Y.-H.L.); jhzhang429@163.com (J.-H.Z.)

**Keywords:** gene vectors, Michael addition, lipopolymers, steroids

## Abstract

Rapid enzymatic degradation and fragmentation during DNA administration can result in limited gene expression, and consequently, poor efficacy. It is necessary to use novel vectors for DNA delivery. Herein, we aimed to design useful carriers for enhancing transfection efficiency (TE). These lipopolymers were prepared through Michael addition reactions from low-molecular-weight (LMW) polyethyleneimine (PEI) and linkers with three kinds of steroids. Agarose gel electrophoresis assay results displayed that the three lipopolymers could condense plasmid DNA well, and the formed polyplexes had appropriate sizes around 200–300 nm, and zeta potentials of about +25–40 mV. The results of in vitro experiments using HeLa, HEK293, and MCF-7 cells showed that these lipopolymers present higher TE than 25-kDa PEI, both in the absence and presence of 10% serum. Flow cytometry and confocal microscopy studies also demonstrated that these lipopolymer/DNA complexes present higher cellular uptake and intracellular distribution. The measurement of critical micelle concentration (CMC) revealed that these lipopolymers could form micelles, which are suited for drug delivery. All results suggest that the three materials may serve as hopeful candidates for gene and drug delivery in future in vivo applications.

## 1. Introduction

Currently, cancer is the main cause of death worldwide. Radiation therapy, chemotherapy, and surgery are the three prime means of treatment [1,2]. Unfortunately, these treatment methods induce strong side effects and lead to terrible sequelae or relapse [3]. Gene therapy is used as a new-generation strategy to cure intractable diseases and genetic disorders that plague civilization [4,5]. Gene delivery vectors can be segmented into viral and non-viral vectors. The share of viral approaches in total clinical trials remains high [6]; however, acute immune response, immunogenicity, insertion mutagenesis, and issues related to the production of viral vectors imposed serious limits on their use [7]. The past two decades witnessed a climax in the development of non-viral vectors for gene therapy [8]. Their advantages involve low immune responses, inexpensive syntheses on a large scale, and high flexibility regarding the delivery of larger sizes of genes [9]. As for non-viral vectors, various natural or synthetic cationic polymers were studied, such as polyethyleneimine (PEI), poly(l-lysine), polyamidoamine (PAMAM), and chitosan [10]. Among these materials, PEI, in particular high-molecular-weight (HMW) branched 25-kDa PEI, is applied as the gold standard for non-viral gene transfection at present [11,12,13,14]. However, complex aggregation on the cell surface, which is caused by strong cationic charge density, results in severe cytotoxicity and the induction of necrosis [15,16]. One alternative way of lowering the toxicity of PEI is to use low-molecular-weight (LMW) PEIs; however, they also cannot be used as gene vectors due to their low transfection efficiency (TE). LMW (0.6 to 2.0 kDa) PEIs were used as starting templates for preparing effective vectors by chemically decorating them using techniques such as electrostatic coating [17], cross-linking [18], and covalent grafting [19]. Cross-linked LMW PEIs with biodegradable ester were widely researched for in vitro transfection over the past decades [20,21,22,23,24]. It was demonstrated that these materials can improve TE and reduce cytotoxicity compared to 25-kDa PEI.

As the first step to entering cells, the polyplexes must traverse through a hydrophobic lipid plasma membrane [25]. Hydrophobic modification usually increases the efficiency of PEIs by virtue of improving cellular uptake [26,27,28]. These substitutions produce relatively innoxious PEI derivatives with excellent efficacy in gene delivery. Our group studied a range of LMW PEI-based biodegradable lipopolymers with different hydrophobic chains, such as fatty acids, and the effect of the long chains on gene delivery was discussed [29].

Based on the polymers of Reference [29], in this report, other hydrophobic chains involving steroids, such as α-tocopherol (vitamin E), cholesterol, and diosgenin, which are biocompatibile, were used as hydrophobic linkers to cross-link LMW PEIs to improve their TE. On one hand, compared with fatty-acid chains, steroids also have active membrane fusion, but they present rigidity, and their own internal cyclic structure makes the formed lipopolymers more stable. Moreover, hydrophobic chains and ester bonds are in the same bridged compound, which avoids the cross-linking of hydrophobic chain linkers and biodegradable ester-bond linkers. Because the cross-linking needs to filtrate the proportion of hydrophobic chain linkers and ester-bond linkers, the proportion of them needs to be calculated to ensure they react on the basis of feed ratio.

## 2. Materials and Methods

### 2.1. Materials

All reagents were obtained commercially and used as received. Anhydrous CH_2_Cl_2_ was purified using standard methods. Branched LMW PEI 600 was obtained from Aladdin, Shanghai, China, while branched PEI 25 kDa was obtained from Sigma-Aldrich, St. Louis, MO, USA. Label IT^®^ Cy5^TM^ was provided by Mirus, Madison, WI, USA; pGL-3 was provided by Promega, Madison, WI, USA; pEGFP-N1 (coding for Enhanced Green Fluorescent Protein (EGFP) DNA) was provided by Clontech, Palo Alto, CA, USA. Dulbecco’s modified Eagle’s medium (DMEM) and fetal bovine serum (FBS) were obtained from Invitrogen Corp (Carlsbad, CA, USA). The MicroBCA protein assay kit was obtained from Pierce, Rockford, IL, USA, while the luciferase assay kit was provided by Promega, Madison, WI, USA. The endotoxin-free plasmid purification kit was obtained from TIANGEN, Beijing, China.

^1^H-NMR spectra were measured on a Bruker AV400 spectrometer. The molecular weight (Mw) was measured using gel permeation chromatography (GPC), with a Waters 515 pump, a Waters 2410 Refractive Index Detector (25 °C, incorporating Shodex columns OHPAK KB-803, Santa Barbara, CA, USA). The mobile phase involved 0.5 mol·L^−1^ HAc/NaAc buffer at a flow rate of 0.5 mL·min^−1^; poly(ethylene glycol) was used as the standard (average Mw: 900–80,000 Da).

### 2.2. Synthesis and Characterization of ***6a***–***6c***

Compound **3** was prepared on the basis of our previously reported procedure [21]. Then, trifluoroacetic acid solution was added dropwise to **3**, and the reaction mixture was stirred overnight at room temperature. The solvent was removed under reduced pressure, and the residue was washed with diluted NH_3_·H_2_O to alkalinity, affording **4**. In addition, **5a**–**5c** were synthesized as follows: steroids (α-tocopherol, cholesterol, and diosgenin; 0.012 mol) and triethylamine (0.012 mol) were dissolved in 50 mL of anhydrous CH_2_Cl_2_ in an ice bath, before triphosgene (0.004 mol) in anhydrous dichloromethane was added dropwise to the solution. The mixture was stirred overnight at room temperature, and the crude product was purified with silica gel column chromatography (petroleum ether, PE). Then, **5a**–**5c** (0.02 mol), **4** (0.022 mol), and 4-dimethylaminopyridine (DMAP, 0.022 mol) were dissolved in 50 mL of anhydrous dichloromethane, and stirred overnight at room temperature. The crude product was purified with silica gel column chromatography (PE:EA (ethyl acetate) = 2:1) to give **6a**–**6c**.

**6a** (yield 14.6%): ^1^H NMR (400 MHz, CDCl_3_): δ = 0.84–0.88 (m, 12H, tocopherol *CH*_3_), δ = 1.07–1.79 (m, 26H, tocopherol H,) δ = 2.00 (s, 3H, tocopherol Ph*CH*_3_), δ = 2.03 (s, 3H, tocopherol Ph*CH*_3_), δ = 2.08 (s, 3H, tocopherol Ph*CH*_3_), δ = 2.57–2.60 (t, *J* = 6.8 Hz, 2H, tocopherol Ph*CH*_2_), 3.71–3.74 (t, *J* = 5.4 Hz, 2H, (OCH_2_*CH*_2_N)_2_), δ = 3.82–3.85 (t, *J* = 5.8 Hz, 2H, (OCH_2_*CH*_2_N)_2_), δ = 4.38–4.41 (t, *J* = 5.8 Hz, 2H, (O*CH*_2_CH_2_N)_2_), δ = 4.44–4.47 (t, *J* = 5.8 Hz, 2H, (O*CH*_2_CH_2_N)_2_), δ = 5.85–5.89 (dd, *J* = 1.2, 10.4 Hz, 2H, (CH_2_C*H*CO)_2_), δ = 6.11–6.19 (ddd, *J* = 4.8, 10.4, 17.2 Hz, 2H, (C*H*_2_CHCO)_2_), δ = 6.41–6.47 (dd, *J* = 1.2, 17.2 Hz, 2H, (C*H*_2_CHCO)_2_); ^13^C NMR (100 MHz, CDCl_3_) δ 166.0 (2C), 154.6, 149.4, 140.8, 131.6, 131.4, 128.2, 128.1, 127.3, 125.5, 123.1, 117.5, 75.1, 62.9, 62.3, 47.8, 47.4, 39.5, 37.7, 37.6, 37.5 (2C), 37.4, 32.9 (2C), 28.1, 24.9, 24.6, 22.9, 22.8, 21.2, 20.7, 19.9, 19.8 (2C), 19.7, 13.1, 12.3, 11.9; high-resolution mass spectrometry (HRMS; electrospray ionization (ESI)): *m*/*z* 692.4504 ([M + Na]^+^), C_40_H_63_NO_7_Na^+^, calculated 692.4502.

**6b** (yield 22.1%): ^1^H NMR (400 MHz, CDCl_3_): δ = 0.67 (s, 3H, cholesterol *CH*_3_), δ = 0.85–1.56 (m, 33H, cholesterol H), δ = 1.80–2.04 (m, 5H, cholesterol H), δ = 2.28–2.38 (m, 2H, cholesterol H), δ = 3.57–3.61 (m, 4H, (OCH_2_*CH*_2_N)_2_), δ = 4.25–4.32 (m, 4H, (O*CH*_2_CH_2_N)_2_), δ = 4.48–4.54 (m, 1H, cholesterol COO*CH*(CH_2_)_2_), δ = 5.35–5.37 (m, 1H, cholesterol C=*CH*), δ = 5.83–5.87 (dd, *J* = 3.2, 10.4 Hz, 2H, (CH_2_C*H*CO)_2_), δ = 6.08–6.15 (dd, *J* = 10.4, 17.6 Hz, 2H, (C*H*_2_CHCO)_2_), δ = 6.39–6.43 (d, *J* = 17.2 Hz, 2H, (C*H*_2_CHCO)_2_); ^13^C NMR (100 MHz, CDCl_3_) δ 166.1, 155.7, 139.8, 131.5, 131.3, 128.3 (2C), 122.8 (2C), 75.5, 62.9, 62.7, 56.8, 56.3, 50.1, 47.4, 47.0, 42.4, 39.9, 39.7, 38.7, 37.1, 36.7, 36.3, 35.9, 32.0 (2C), 28.4, 28.3, 28.2, 24.4, 24.0, 23.0, 22.7, 21.2, 19.5, 18.9, 12.0; HRMS (ESI): *m*/*z* 648.4239 ([M + Na]^+^), C_38_H_59_NO_6_Na^+^, calculated 648.4240.

**6c** (yield 25.3%): ^1^H NMR (400 MHz, CDCl_3_): δ = 0.72–0.73 (m, 6H, diosgenin *CH*_3_), δ = 0.90–1.95 (m, 28H, diosgenin H), δ = 2.19–2.34 (m, 2H, diosgenin H), δ = 3.28–3.33 (AB, A of AB, *J*_1_ = *J*_2_ = 10.8 Hz, 1H, diosgenin O*CH*_2_), δ = 3.39–3.41 (AB, B of AB, *J* = 2.0, 10.4 Hz, 1H, diosgenin O*CH*_2_), δ = 3.51–3.54 (m, 4H, (OCH_2_*CH*_2_N)_2_), δ = 4.21–4.25 (m, 4H, (O*CH*_2_CH_2_N)_2_), δ = 4.31–4.37 (td, *J* = 7.6 Hz, 1H, diosgenin CO*CH*), δ = 4.42–4.47 (m, 1H, diosgenin COO*CH*(CH_2_)_2_), δ = 5.30–5.31 (m, 1H, diosgenin C=*CH*), δ = 5.77–5.80 (dd, *J* = 3.2, 10.4 Hz, 2H, (CH_2_C*H*CO)_2_), δ = 6.03–6.10 (dd, *J* = 10.4, 17.2 Hz, 2H, (C*H*_2_CHCO)_2_), δ = 6.32–6.37 (d, *J* = 17.6 Hz, 2H, (C*H*_2_CHCO)_2_); ^13^C NMR (100 MHz, CDCl_3_) δ 165.8, 155.5, 139.7, 131.3, 131.1, 128.2, 128.1, 122.3, 109.2, 80.8, 75.2, 66.8, 62.7, 62.5, 62.1, 56.4 (2C), 49.9, 47.3, 46.9, 41.6, 40.2, 39.7, 38.5, 36.9, 36.7, 32.0, 31.8, 31.4 (2C), 30.3, 28.8, 28.1, 20.8, 19.4, 17.2, 16.3, 14.5; HRMS (ESI): *m*/*z* 676.3829 ([M + Na]^+^), C_38_H_55_NO_8_Na^+^, calculated 676.3825.

### 2.3. Synthesis and Characterization of ***7a***–***7c***

Lipopolymers were synthesized as follows: PEI 600 (1.26 mmol) and linkers **6a**–**6c** (1.26 mmol) were dissolved in 3 mL of anhydrous CH_2_Cl_2_ under N_2_ atmosphere; then, the mixtures were heated at 45 °C with constant stirring in an oil bath for 72 h. The crude product was dissolved in 0.5 mL of anhydrous dimethyl sulfoxide (DMSO), before being precipitated by anhydrous (CH_3_CH_2_)_2_O. The precipitation was dried in vacuum to obtain the colorless oil product. The Mw of lipopolymers **7a**–**7c** were detected by GPC. The yields of **tocopherol**–**PEI**, **cholesterol**–**PEI**, and **diosgenin**–**PEI** were 54.03%, 51.27%, and 50.33%, respectively. For detailed peak assignments, see (Figure S1) (Supplementary Materials).

### 2.4. Buffer Capacity Test

Lipopolymer (amino groups: 0.25 mmol) was dissolved in 5 mL of NaCl aqueous solution (150 mM); then, HCl (1 M) was added to adjust the pH to 2.0. Fifty milliliters of NaOH (0.1 M) was added to each solution; after each addition, the pH of the solution was measured with a pH meter (pHS-25). The 25-kDa PEI polymer was used as a control under the same experimental conditions. The buffer capacity was interpreted as the percentage of amine groups (protonated from pH 5.1 to 7.4), using Equation (1).

buffer capacity (%) = 100 × (ΔV_1_NaOH − ΔV_2_NaOH) × 0.1 M/Nmol, (1)

where ΔV_1_NaOH is the volume of 0.1 M NaOH solution for bringing the pH of the lipopolymer solution from 5.1 to 7.4, ΔV_2_NaOH is the volume of 0.1 M NaOH solution for bringing the pH of the NaCl solution from 5.1 to 7.4, and N is the total number of moles (0.25 mmol) of protonable amine groups in the lipopolymer.

### 2.5. Measurement of Critical Micelle Concentration

The critical micelle concentration (CMC) was measured using pyrene as a fluorescence probe. The lipopolymer concentration was transformed from 1.0 × 10^−6^ mg/mL to 1 mg/mL, while the pyrene concentration was fixed at 6.0 × 10^−7^ M. The fluorescence spectra were recorded using a FluoroMax-4 fluorescence spectrometer. The emission and excitation slit widths were 5 nm. The samples were excited at 335 nm and the emission spectra were recorded from 350 to 500 nm. The emission fluorescence values *I*_373_ (373 nm) and *I*_383_ (383 nm) were used for the subsequent calculations. The CMC value was measured from the plots of the *I*_383_/*I*_373_ ratio versus the logarithm of the lipopolymer concentration using the intersection of the linear regression lines.

### 2.6. Cell Culture

U2OS cells, HEK293 cells, MCF-7 cells, HepG2 cells, and HeLa cells were incubated in DMEM containing 1% antibiotics (penicillin–streptomycin, 10,000 U·mL^−1^) and 10% FBS (37 °C, humidified atmosphere with 5% CO_2_).

### 2.7. Amplification and Purification of pEGFP-N1 and pGL-3 Plasmids

The pGL-3 plasmids were transformed in *Escherichia coli* JM109, while the pEGFP-N1 plasmids were transformed in *E. coli* DH5α. Both plasmids were amplified at 37 °C in Luria-Bertani broth overnight, before being purified with an EndoFree Tiangen TM Plasmid Kit. The purified plasmids were dissolved in TE buffer solution and then stored at −20 °C. The integrity of the plasmids was determined via agarose gel electrophoresis. The purity and concentration of the plasmids were confirmed using their ultraviolet absorbance at 280 and 260 nm.

### 2.8. Agarose Gel Electrophoresis

Lipopolymer/DNA complexes were prepared at *w*/*w* concentrations of 0.1, 0.2, 0.4, 0.8, 1.6, 3.2, and 6.4 (pUC 19: 0.025 mg/mL, 5 µL). Then, the complex solutions were diluted to 15 µL, after which they were incubated at 37 °C for 30 min, the complexes were electrophoresed on a 1% (*w*/*v*) agarose gel including GelRed^TM^ (Biotium, Bay Area, State of CA, USA) and Tris-acetate running buffer at 150 V for 30 min. DNA was visualized under an ultraviolet lamp using a Vilber Lourmat imaging system (Biorad universal hood II, Biorad, Hercules, CA, USA).

### 2.9. Ethidium Bromide Displacement

Ethidium bromide (EB) exclusion assays were used to present the ability of the lipopolymer to condense DNA. Fluorescence spectra were measured using a Horiba Jobin Yvon Fluoromax-4 spectrofluorometer. Briefly, 5 µL of 1 mg/mL EB was dissolved in 2.5 mL of 4-(2-hydroxyethyl)-1-piperazineethanesulfonic acid (HEPES) solution (10 mM). The fluorescence intensity of EB was detected after shaking. Then, 10 µL of 1 mg/mL circulating tumor DNA (ctDNA) was added, after which the fluorescence intensity of the interaction between DNA and EB was measured after shaking. Subsequently, 0.2 mg/mL lipopolymer solution (5 µL for each addition) was added for further measurement. The excitation and emission spectra were measured at 520 nm and 600 nm, respectively. Pure EB solution and DNA/EB solution were used as negative and positive controls, respectively. The percentage relative fluorescence (%F) was confirmed using Equation (2).

%F = (F − F_EB_)/(F_0_ − F_EB_), (2)

where F_EB_ is the fluorescence intensity of the pure EB solution, and F_0_ is the fluorescence intensity of the DNA/EB solution.

### 2.10. Measurements of Particle Size and Zeta Potential in Water

The particle sizes and zeta potentials of lipopolymer/DNA polyplexes (1 µg pUC 19; *w*/*w* concentrations of 0.2, 0.4, 0.8, 1.6, 3.2, and 6.4) were measured using a Nano-ZS 3600 (Malvern Instruments, Malvern, Worcestershire, UK) with a He–Ne laser beam (633 nm) at 25 °C. The polyplexes were incubated at 37 °C for 30 min, after which they were diluted with deionized water to 1 mL. Data are displayed as means ± standard deviation (SD) on account of triplicate independent experiments. In addition, the lipopolymer solutions were prepared at concentrations of 1 mg/mL and 3 mg/mL, before which their sizes (zeta) and morphologies were detected using dynamic light scattering (DLS) and transmission electron microscopy (TEM, Hitachi High-Tech Science, Schaumburg, IL, USA), respectively.

### 2.11. Cell Viability

The toxicity of lipopolymers toward HeLa cells, and the cell viability of the complexes using HEK293 cells and MCF-7 cells were detected using a Cell Counting Kit-8. The HeLa cells and HEK293 cells (8000 cells/well), and the MCF-7 cells (9000 cells/well) were seeded into 96-well plates, and then cultured until 70–80% cell confluence. The cells were incubated in a culture medium including lipopolymer with different concentrations for 24 h (complexes: 0.2 μg pEGFP). Then, the lipopolymer solution was replaced by 100 μL of 0.1 mg/mL sterile filtered CCK-8 stock solution in phosphate-buffered saline (PBS) for another hour of incubation at 37 °C. Then, the absorbance of each sample was detected using an ELISA plate reader (model 680, BioRad, Hercules, CA, USA, 450 nm). Cell viability was confirmed using Equation (3), and 25-kDa PEI was used as a control.

cell viability = (OD_treated_/OD_control_) × 100%, (3)

where OD is the optical density.

### 2.12. Transfection Assay

The gene transfection of complexes toward HeLa, HEK293, MCF-7, HepG2, and U2OS cells was studied. Firstly, 1.1 × 10^5^ cells/well of U2OS, MCF-7, and HepG2 cells, and 9 × 10^4^ cells/well of HEK293 and HeLa cells were seeded in 24-well plates, and then cultured until 70–80% cell confluence. The medium was replaced by 10%-serum-containing or serum-free culture medium, which contained lipopolymer/plasmid DNA (pDNA) complexes (0.8 µg) at various *w*/*w* concentrations. After 4 h of incubation at 37 °C in 5% CO_2_, the medium was replaced by a 10%-serum-containing culture medium, and then incubated for another 20 h.

For enhanced green fluorescent protein (EGFP) assays, GFP-expressed cells were observed 24 h post transfection using an inverted fluorescence microscope (Nikon Eclipse TE 2000E, Nikon, Sendai, Japan) and digested using trypsin for flow cytometry analysis (BD FACSCanto II, BD Biosciences, New York, NY, USA).

For the cells transfected with pGL-3, after 24 h of incubation as described above, the cells were washed using cold PBS and then lysed using lysis reporter buffer (Promega, 100 µL). The luciferase activity of the lysed cell was detected using a microplate reader (Model 550, BioRad, Hercules, CA, USA). Protein content was detected using a bicinchoninic acid (BCA) protein assay. Gene TE was considered as the relative fluorescence intensity (relative light units) per mg of protein (RLU/mg protein) on the basis of experiments done in triplicate.

### 2.13. Cellular Uptake

The cellular uptake of complexes was detected with flow cytometry. A Label IT Cy5 Labeling Kit was used to label pDNA based on the manufacturer’s protocol. HEK293 and HeLa cells were seeded in 12-well plates (2.0 × 10^5^ cells/well) and cultured for 24 h. The medium was replaced by serum-free medium with Cy5-labeled DNA complexes (1.6 μg of DNA, optimal *w*/*w*). After 4 h of incubation at 37 °C in 5% CO_2_, the cells were washed with PBS, and then harvested with 0.25% trypsin/ethylenediaminetetraacetic acid (EDTA), before finally being resuspended in PBS. Mean fluorescence intensity was determined using a flow cytometer (BD FACSCanto II, BD Biosciences, New York, NY, USA). Cy5-labeled DNA was tested on the FL-4 channel (633 nm). The flow cytometer was standardized to get a background level of ~1% for the control sample.

### 2.14. Confocal Laser Scanning Microscopy

HeLa cells were seeded in a 35-mm confocal dish (Φ = 15 mm, 1.5 × 10^4^ cells/well) and cultured for 24 h. The medium was replaced by serum-free or 10%-serum-containing medium, which contained Cy5-labeled DNA complexes (0.8 μg of DNA, optimal *w*/*w*). After 4 h of incubation at 37 °C in 5% CO_2_, the cells were washed twice with PBS (pH 7.4), and fixed by 4% paraformaldehyde (dissolved in PBS buffer) for 10 min; then, their nuclei were stained using 4′,6-diamidino-2-phenylindole (DAPI). Confocal laser scanning microscopy (CLSM, Leica, Solms, Germany) observations were carried out using a Leica TCS SP5 at excitation wavelengths of 633 nm for Cy5 (red) and 405 nm for DAPI (blue).

## 3. Results and Discussion

### 3.1. Synthesis and Chemical Properties of Target Lipopolymers

**Scheme 1:**
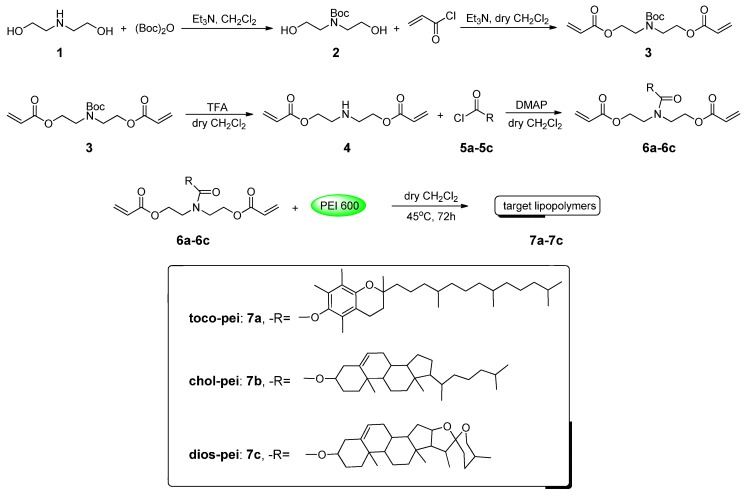
Synthetic steps for lipopolymers.

In this study, we used biocompatible steroid linkers to cross-link LMW PEI, as shown in Scheme 1. The amino in the middle of bis(β-hydroxylethyl)amine was first protected by (Boc)_2_O, and two equivalents of acryloyl chloride were added to form compound **3**; then, compound **4** was obtained in the presence of trifluoroacetic acid and subsequent ammonium hydroxide lavation. Compounds **6a**–**6c** were prepared by mixing compound **4** and steroid-containing **5a**–**5c**. Then, PEI 600 Da and the linkers **6a**–**6c** (molar ratio of 1:1) were polymerized in anhydrous dichloromethane at 45 °C for 72 h via a Michael addition reaction. The crude products were precipitated with dimethyl sulfoxide and anhydrous diethyl ether three times to ensure their polydispersity. GPC was used to test the Mw of the lipopolymers, as shown in Table 1. The molecular weights of the target compounds were not very high due to their large steric hindrance, and the Mw of the three lipopolymers had no distinct difference, which might also be due to the hindrance.

**Figure 1:**
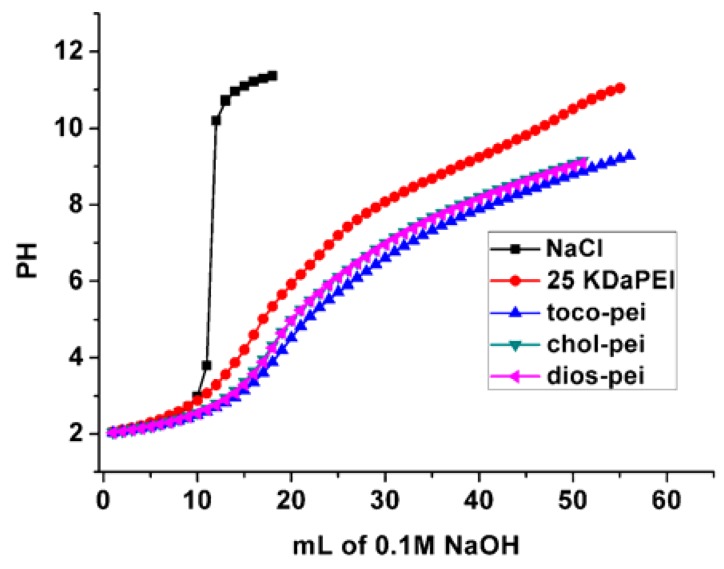
Acid–base titration profiles.

Acid–base titration was performed to evaluate the proton-buffering effect of the cationic polymers. As the gold standard for non-viral gene vectors, 25-kDa PEI is known for its good buffering ability and its “proton-sponge effect”, which might lead to the disruption of the endosome and facilitate the escape of polymer/DNA complexes [30]. In this study, the buffer capabilities of the lipopolymers were evaluated, and the results are shown in Figure 1 and Table 2. The acid–base titration curves in Figure 1 show that all the lipopolymers had a much higher pH-buffering ability than 25-kDa PEI, and the pH range from 7.4~5.1 (endosomal acidification) in Table 2 shows the analogical results. Hydrophobic groups had a trivial effect on the buffer capacity. From the above, it might be implied that the complexes formed by these lipopolymers with DNA could easily escape from the endosome, which is beneficial for gene delivery.

**Figure 2:**
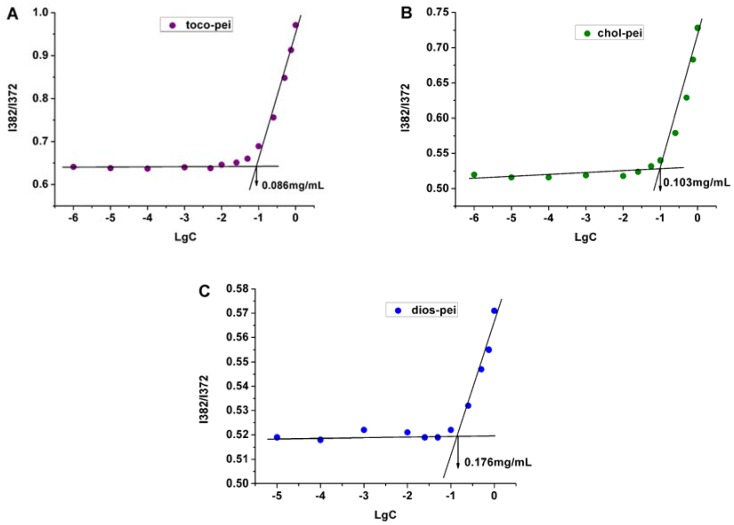
Plots of intensity (*I*_383_/*I*_373_) ratios from the emission spectra of pyrene versus the logarithm of the three lipopolymer concentrations in aqueous solution: (**A**) Tocopherol–polyethyleneimine (PEI; **toco-pei**); (**B**) cholesterol–PEI (**chol-pei**); (**C**) diosgenin–PEI (**dios-pei**).

The lipopolymers are amphiphilic in nature, because PEI is hydrophilic, while the steroids are hydrophobic. With an increase in concentration, the lipopolymers might form multimolecular micelles or micellar aggregates in water. The CMC of these lipopolymers was detected using pyrene as a fluorescence probe [31,32]. Figure 2 depicts the intensity ratios (*I*_383_/*I*_373_) of the pyrene against the logarithm of lipopolymer concentrations. The CMC values of the three different lipopolymers ranged from 0.086 to 0.176 mg/mL, and they increased with increasing molecular weight.

**Figure 3:**
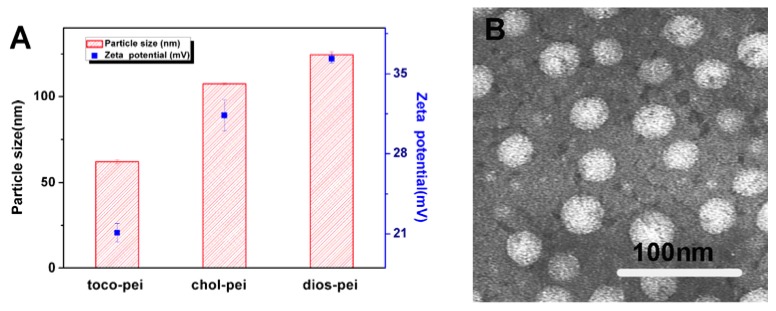
Particle sizes and zeta potentials of the three lipopolymers (**A**); TEM image of **dios-pei**, 80,000× magnification, 75 kV (**B**).

Particle size affects the biodistribution and circulation time of gene vectors in vivo. The particles with an average size below 200 nm can decrease uptake by the reticuloendothelial system, and they offer a passive tumor-targeting power by means of the retention effect and an enhanced permeability [33,34]. In this report, TEM and DLS measurements were carried out to confirm the micelle morphologies and sizes. Figure 3A shows that the average sizes of the micelles were 62.28 nm, 107.50 nm, and 124.53 nm for tocopherol–PEI (**toco-pei**), cholesterol–PEI (**chol-pei**), and diosgenin–PEI (**dios-pei**), respectively. According to the TEM images (Figure 3B), the micelles of **dios-pei** adopted a spherical morphology, and their size was about 20–30 nm. Microscopy for particle size is considered as an absolute measurement, and it can separate aggregates from single particles. DLS is used to confirm size distribution. Therefore, the particle size determined using DLS is larger than that using microscopy [35]. There is also an important influence of zeta potential on the stability of a colloid dispersion system. As seen in Figure 3A, all the blank lipopolymers had positive charges because of the protonated PEI, facilitating their electrostatic interaction with DNA. Combining this result with the TEM image, it can be seen that electrostatic repulsion between the micelles did not take place.

### 3.2. Properties of Lipopolymer/DNA Polyplexes

**Figure 4:**
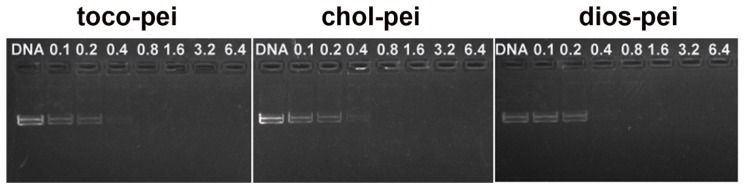
Agarose gel electrophoresis, from left to right: plasmid DNA (pDNA), and lipopolymer/DNA complexes with *w*/*w* concentrations of 0.1, 0.2, 0.4, 0.8, 1.6, 3.2, and 6.4.

For cationic polymer-mediated gene transfections, the first step is the electrostatic interaction between carrier/DNA polyplexes and the negative plasma membrane [36]. To study the electrostatic interaction between the lipopolymers and DNA, an agarose gel electrophoresis retardation assay was performed. As shown in Figure 4, the lipopolymers could completely inhibit the mobility of DNA at *w*/*w* concentrations higher than 0.8. At the *w*/*w* concentration of 0.4, free DNA migrated for **toco-pei** and **chol-pei**, but not for **dios-pei**, which might be due to the relatively higher molecular weight of **dios-pei**. In short, they all could bind plasmid DNA well and facilitate interaction with the anionic plasma membrane in the first transfection step.

**Figure 5:**
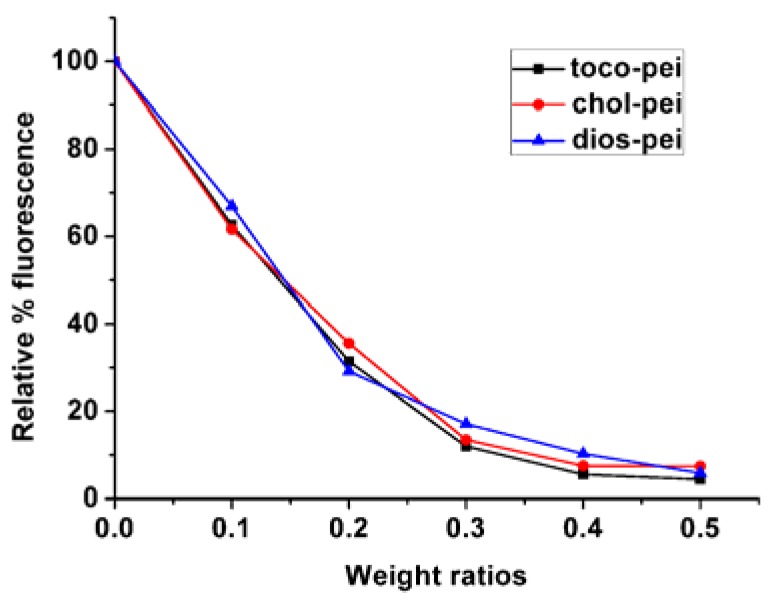
Fluorescence quenching assay using ethidium bromide (EB)/DNA followed by addition of lipopolymers.

The affinity of the lipopolymers for DNA was also determined using a fluorescence quenching test with EB. EB has weak fluorescence; however, in the presence of DNA, its emission intensity can be enhanced due to the strong intercalation between the adjacent DNA base pairs. This enhanced fluorescence could be quenched by the addition of a DNA-binding molecule with higher affinity; thus, the decreased fluorescence intensity can be used as a measurement for DNA complexation efficiency [37]. The results are shown in Figure 5; with the addition of the lipopolymers, the fluorescence intensity of EB decreased rapidly. At *w*/*w* ratios of 0.4 or above, the relative fluorescence intensity became approximately stable, indicating a nearly full DNA condensation. These results are in agreement with those gained from the gel retardation test.

**Figure 6:**
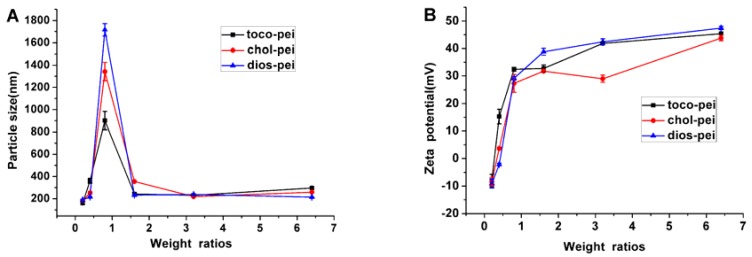
Average particle sizes (**A**) and average zeta potentials (**B**) of lipopolymer/DNA polyplexes at *w*/*w* concentrations of 0.2, 0.4, 0.8, 1.6, 3.2, and 6.4 (mean ± SD, *n* = 3).

The size and surface charge of the complexes were deemed as important parameters [38] for gene delivery, because they dramatically influence the TE, cytotoxicity, and intracellular uptake of the nano-scaled payloads [39,40]. Herein, the zeta potentials and particle sizes of lipopolymer/DNA polyplexes were tested at different *w*/*w* ratios using DLS. With an increase in *w*/*w* ratio, the particle sizes of the polyplexes tended to be steady with a range of 200–300 nm (Figure 6A). In general, the particles formed with sizes less than 350 nm beyond a *w*/*w* ratio of 1.6, at which point full retardation was noticed in the gel electrophoresis. It demonstrated that the type of steroid and the Mw of the lipopolymers had a slight effect on the size. The zeta potential values increased with an increase in lipopolymer concentration (DNA concentration was fixed in the mass ratio) (Figure 6B). However, beyond the weight ratio of 1.6, there was no further change despite the increase in lipopolymer concentration. Among these complexes, **chol-pei**/DNA showed the lowest zeta potential, which might be due to the positive charge of this complex being screened by hydrophobic cholesterol.

### 3.3. Cytotoxicity

**Figure 7:**
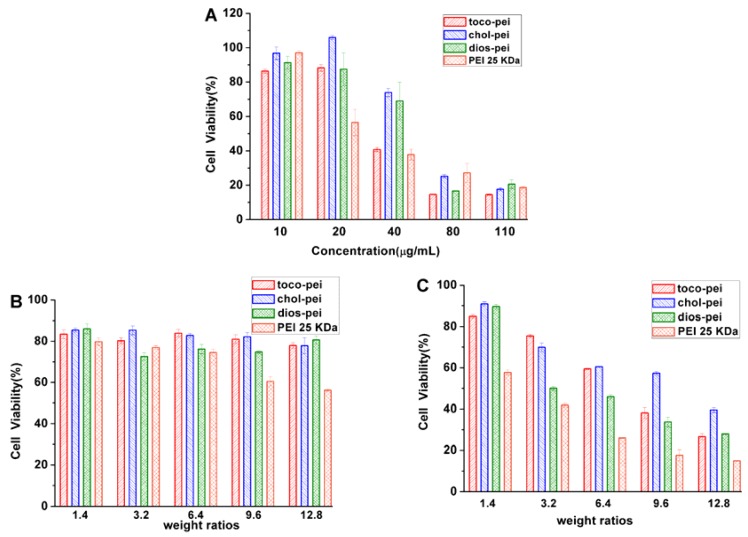
Cell viabilities of lipopolymers at all kinds of concentrations toward HeLa cells (**A**), and lipopolymers/plasmid enhanced GFP (pEGFP) polyplexes at different *w*/*w* ratios toward MCF-7 (**B**) and HEK293 cells (**C**); 25-kDa PEI was used as the control (mean ± SD, *n* = 3).

A successful delivery system should present high TE and low toxicity. The cell viability of the lipopolymers at all kinds of concentrations (including the scope used in the gene transfection) toward HeLa cells, as well as the cell viability of lipopolymer/pDNA complexes with different *w*/*w* ratios in the MCF-7 and HEK293 cells, were evaluated using CCK8-based cell viability assays; 25-kDa PEI was used for comparison (Figure 7). From Figure 7A, the cell viability of all lipopolymers was observed to decrease with an increase in concentration. The cytotoxicities of these lipopolymers were similar to that of 25-kDa PEI, which, in addition to the positive charges, might also be due to the membrane fusion ability due to the hydrophobic steroid chains. The cell viabilities of all the lipopolymer/DNA polyplexes remained unchanged with an increase in *w*/*w* ratio toward MCF-7 cells (Figure 7B), while the cytotoxicity of PEI/DNA polyplexes was increased. As for HEK293 cells (Figure 7C), with the increase in *w*/*w* ratio, the cell viability of all the polyplexes was observed to decrease, but the cytotoxicity change of PEI/DNA polyplexes was more dramatic. This might be because the increased number of free polycations, in addition to the polycation/pDNA complexes, induced the increased cytotoxicity [41]. On the other hand, **chol-pei**/DNA polyplexes showed lower cytotoxicity, which might be due to the lower zeta potential of the complexes or the better biocompatibility of cholesterol.

### 3.4. Gene Transfection In Vitro

**Figure 8:**
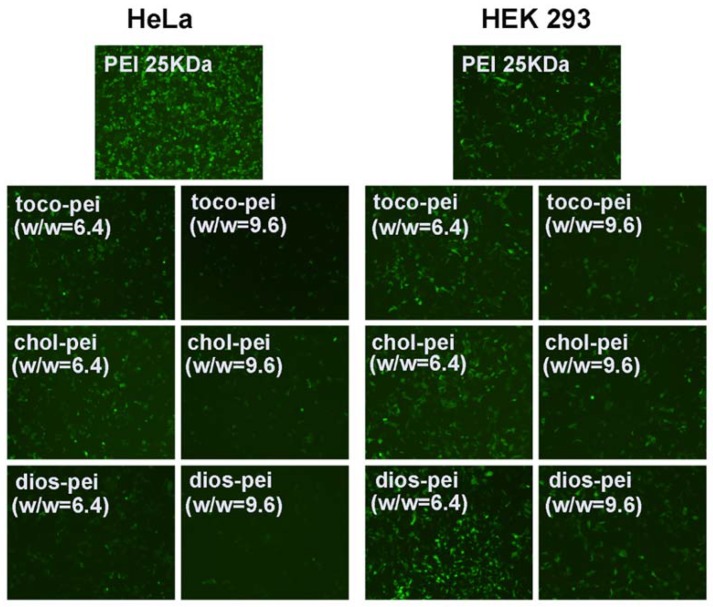
Images of pEGFP-transfected HeLa and HEK293 cells with a serum-free medium at different weight ratios (*w*/*w* = 6.4 or 9.6); 25-kDa PEI was used for comparison.

**Figure 9:**
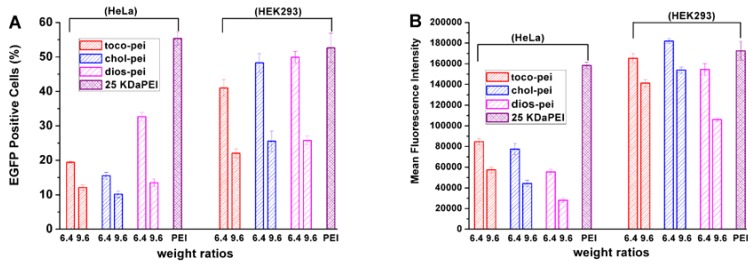
Transfection efficiencies of lipopolymer/DNA polyplexes at different weight ratios (*w*/*w* = 6.4 or 9.6) toward HeLa and HEK293 cells with a serum-free medium; 25-kDa PEI was used for comparison. Data were considered as the percentage of cells expressing (**A**) EGFP-positive cells and (**B**) mean fluorescence intensity (MFI) measured using flow cytometry analysis.

In order to confirm the gene delivery ability of lipopolymer/DNA polyplexes, the direct visualization of gene expression of enhanced green fluorescent protein (EGFP) in HeLa and HEK293 cells was achieved using an inverted fluorescent microscope in the absence of 10% serum. Results in Figure 8 show that the density of transfected cells by the three lipopolymer/DNA polyplexes was lower than that obtained from 25-kDa PEI toward HeLa cells, while they performed similarly in HEK293 cells. Subsequently, flow cytometry was carried out, taking both EGFP-positive cells (%) and mean fluorescence intensity (MFI) into consideration. As shown in Figure 9, the proportion and MFI of EGFP-transfected cells were still lower than that of PEI in HeLa cells, while they showed a similar proportion and MFI compared with PEI in HEK293 cells; the results are in accordance with that of Figure 8.

**Figure 10:**
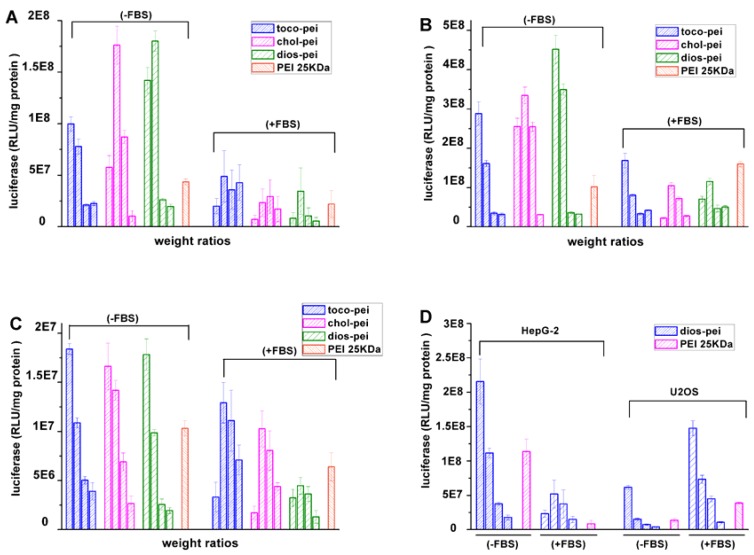
Luciferase expression transfected by lipopolymer/DNA polyplexes at various *w*/*w* ratios (3.2, 6.4, 9.6, or 12.8) in HeLa (**A**), HEK293 (**B**), and MCF-7 (**C**) cells with serum-free or 10% serum-containing medium. (**D**) **dios-pei**/DNA polyplexes at different *w*/*w* ratios (3.2, 6.4, 9.6, or 12.8) in HepG2 and U2OS cells in the absence and presence of 10% serum. All experiments used 25-kDa PEI/pGL-3 (*w*/*w* = 1.4) for comparison (mean ± SD, *n* = 3).

Further in vitro studies were conducted to evaluate the transfection ability of the lipopolymers, and transfection data from the various cell lines are shown in Figure 10. The gold standard 25-kDa PEI with its optimal *w*/*w* of 1.4 was examined in parallel. Figure 10A shows that the luciferase expression levels of all lipopolymer/DNA polyplexes were higher than that of 25-kDa PEI in the absence or presence of 10% serum toward HeLa cells. In particular, **chol-pei**/DNA and **dios-pei**/DNA polyplexes outperformed 25-kDa PEI by factors of 4.03 and 4.12, respectively, with serum-free medium at the optimized *w*/*w* ratio (6.4). However, **toco-pei**/DNA polyplexes showed the highest TE in the presence of 10% serum compared to the other two polyplexes; the same phenomenon was observed in HEK293 cells (Figure 10B) and MCF-7 cells (Figure 10C). It was reported that the α-tocopherol tail and ester-linkage-containing lipid show high serum compatibility [42]; here, the serum tolerance of **toco-pei**/DNA polyplexes might be attributed to the tocopherol and ester contained. Based on the higher TE of **dios-pei**/DNA polyplexes for the three previously described cells, it was selected for evaluation toward HepG2 and U2OS cells in the absence and presence of 10% serum. The results exhibited that **dios-pei**/DNA polyplexes could achieve good gene transfection efficiency in the two cells whether in serum-free or 10%-serum-containing medium, displaying a TE 6.15 times higher than that of 25-kDa PEI toward HepG2 cells with 10% serum-containing medium at the optimized *w*/*w* ratio of 6.4. As for U2OS cells, TEs 4.54 and 3.81 times higher than that of 25-kDA PEI were observed in the absence and presence of 10% serum, respectively. The results indicated that the three lipopolymers have higher TE and might serve as hopeful candidates for gene delivery. The luciferase expression levels of all lipopolymer/DNA polyplexes did not conform with the expression of EGFP, which might be due to the different reporter gene and representing methods.

**Figure 11:**
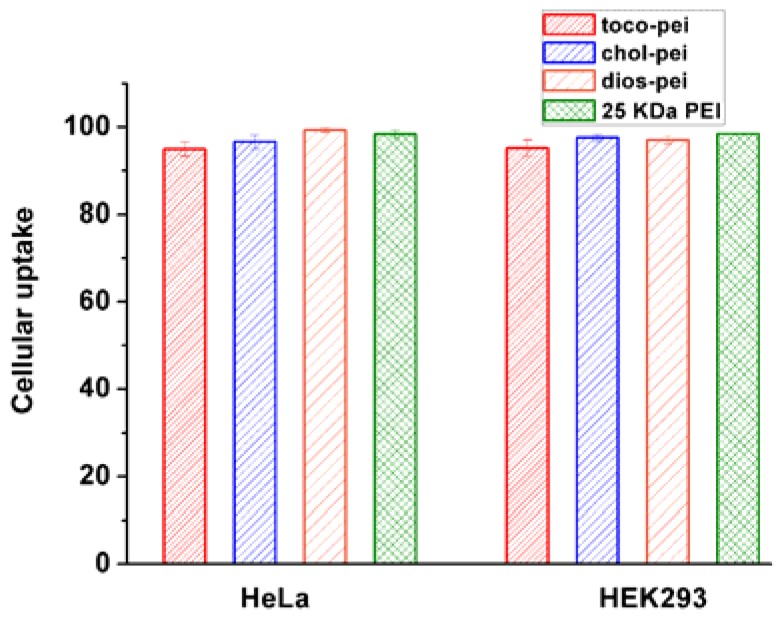
Cellular uptake by flow cytometry analysis: lipopolymer/DNA polyplexes at optimal transfection *w*/*w* ratios toward HeLa and HEK293 cells with a serum-free medium; 25-kDa PEI was used for comparison (mean ± SD, *n* = 3).

The internalization of complexes is one of the primary barriers in gene delivery, and tremendously influences the TE. We used Cy5-incorporated DNA to explore cellular uptake using flow cytometry. After an incubation of 4 h of the lipopolymer/DNA complexes with HeLa cells and HEK293 cells in the absence of serum, the percentage of positive cells for Cy5-labeled pDNA was determined. The results are shown in Figure 11. Whether in HeLa cells or HEK293 cells, all complexes showed cell internalization comparable to that of 25-kDa PEI. The higher cellular uptake might be due to the hydrophobic modifications. Thus, we considered that the better TE might be due to the higher cellular uptake, which agrees with Figure 10A,B.

**Figure 12:**
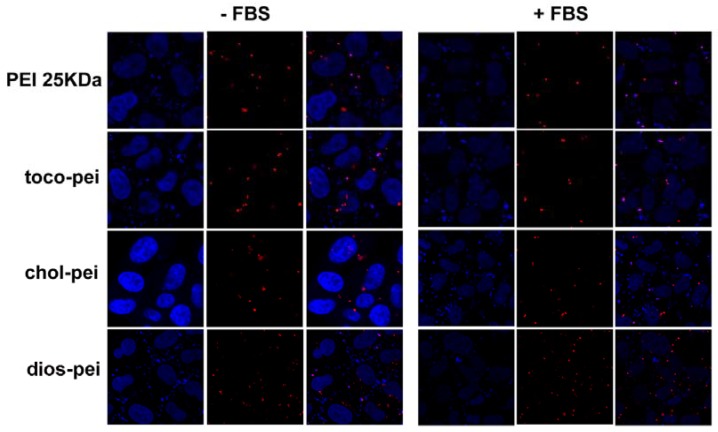
Confocal images toward HeLa cells: lipopolymer/Cy5-labeled DNA polyplexes at optimal transfection *w*/*w* ratios for 4 h; (**left**) serum-free medium; (**right**) 10%-serum-containing medium, 25-kDa PEI was used as a control. For each triplicate image, from left to right: cell nuclei stained by 4′,6-diamidino-2-phenylindole (DAPI; blue); Cy5-labeled pDNA (red); merged image.

The intracellular distribution of transfected DNA was observed toward HeLa cells with serum-free and 10% serum-containing media using confocal laser scanning microscopy. The polyplexes were prepared at the optimal *w*/*w* ratios (Cy5-labeled DNA (red)), and the nuclei were stained with 4′,6-diamidino-2-phenylindole DAPI (blue). As shown in Figure 12, after an incubation of 4 h of complexes with HeLa cells in the absence of serum, a set amount of labeled DNA was delivered to the perinuclear region for lipopolymer/DNA and PEI/DNA complexes, and the difference between them was not obvious, which is in accordance with Figure 11. Similarly, for the 10% serum-containing medium, a small amount of labeled DNA was observed for lipopolymer/DNA and PEI/DNA complexes. In other words, whether in the absence or presence of 10% serum toward HeLa cells, lipopolymer/DNA complexes showed intracellular distribution comparable to that of PEI/DNA, and the higher cellular uptake caused higher TE, which agrees with Figure 10A.

## 4. Conclusions

In this report, three lipopolymers were prepared via Michael addition from linkers with various steroid-containing and LMW PEI complexes. These lipopolymers can condense DNA well, and they form with appropriate zeta potentials and sizes. Their improved buffering capacity can easily result in the disruption of the endosome. In vitro transfection tests revealed that the hydrophobic modified materials have higher TEs than 25-kDa PEI, whether in the absence or presence of 10% serum. Furthermore, these lipopolymers can form micelles, which are suited for drug delivery. All studies suggest that these materials may serve as hopeful candidates for drug and gene delivery.

## Figures and Tables

**Table 1:** Molecular weights (Mw) and polydispersities (PDI) of lipopolymers. PEI—polyethyleneimine.

Compounds	Mw	PDI
**Tocopherol–PEI (toco-pei)**	7880	1.68
**Cholesterol–PEI (chol-pei)**	8974	1.79
**Diosgenin–PEI (dios-pei)**	10,985	1.95

**Table 2:** Buffering capacity.

Sample	toco-pei	chol-pei	dios-pei	25-kDa PEI
Buffer capacity (%)	26.0	24.0	26.0	16.0

## Supplementary Materials

The following are available online at http://www.mdpi.com/2073-4360/10/10/1060/s1: NMR and MS spectra for compounds.
