# Supplementary material for: Synthesis and Properties of Low-Molecular-Weight PEI-Based Lipopolymers for Delivery of DNA
Source: Polymers (Basel). 2018 Sep 25;10(10):1060. doi: 10.3390/polym10101060 (PMC6403936; doi:10.3390/polym10101060)
Supplement: Supplementary file 1 [file polymers-10-01060-s001.pdf]

Electronic Supplementary Information

Synthesis and properties of low molecular weight PEI-based-lipopolymers for delivery of DNA

Miao-Miao Xun*,[a] Zheng Huang,[b] Ya-Ping Xiao,[b] Yan-Hong Liu,[b] Ji Zhang*,[b] Ju-Hui Zhang,[b] Xiao-Qi Yu*[b]

[a]*National Demonstration Center for Experimental Chemical Engineering Comprehensive Education, School of Chemical Engineering and Technology, North University of China, Taiyuan 030000, China*

[b]*Key Laboratory of Green Chemistry and Technology (Ministry of Education), College of Chemistry, Sichuan University, Chengdu 610064, PR China*

*Corresponding authors: mmxun@nuc.edu.cn; xqyu@scu.edu.cn (X.-Q. Yu); jzhang@scu.edu.cn (J. Zhang). Fax: +86-28-85415886 (X.-Q. Yu)

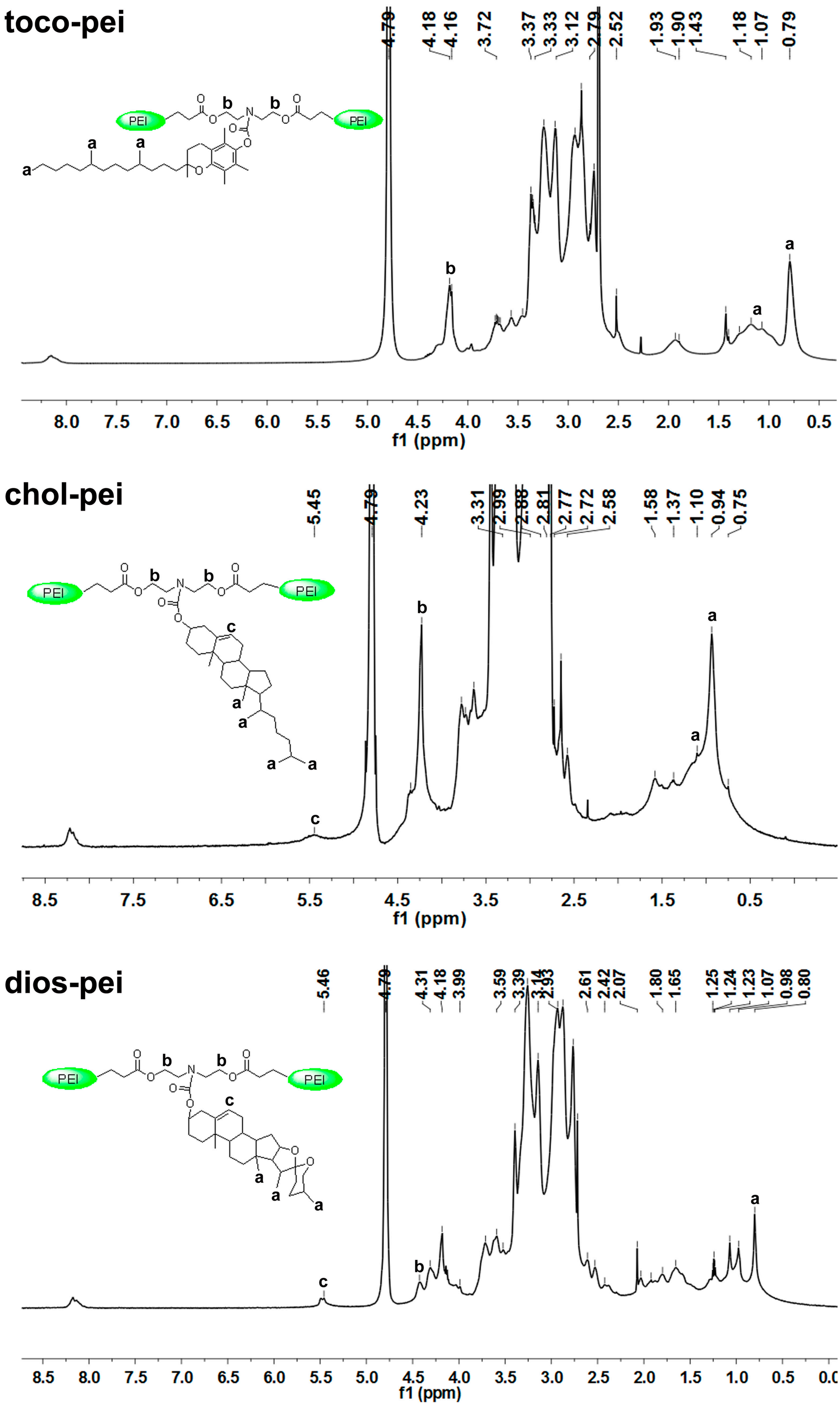

Fig. S1. ^{1}H NMR spectra of the three lipopolymers in D_2O.

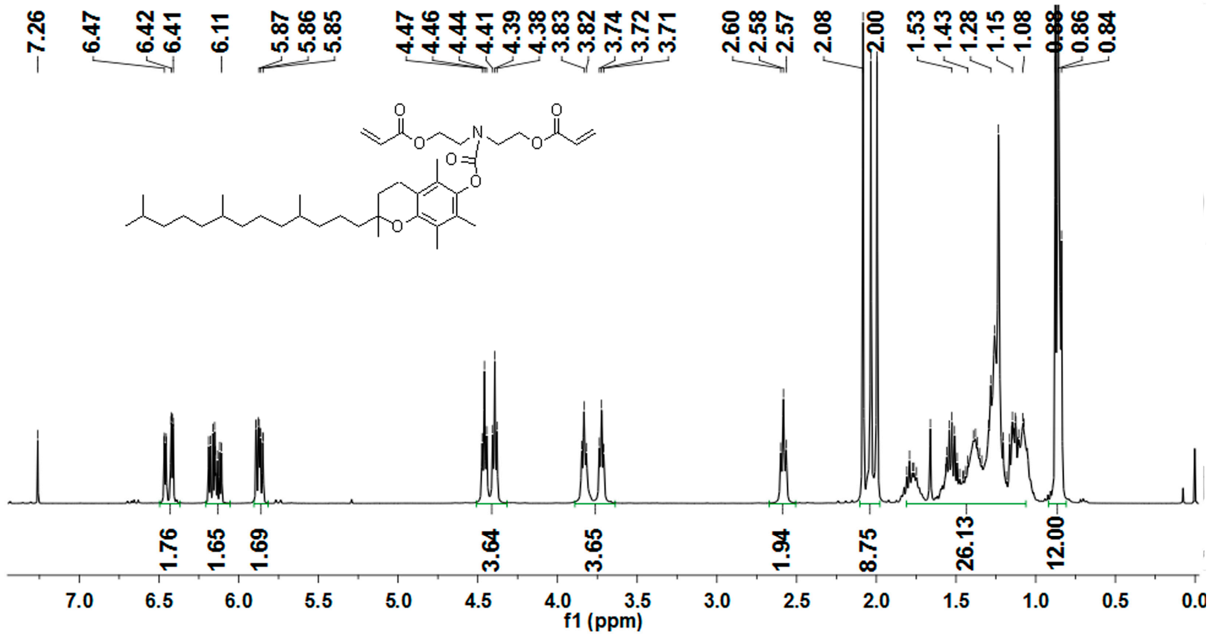

Fig. S2. ^{1}H NMR of **6a.**

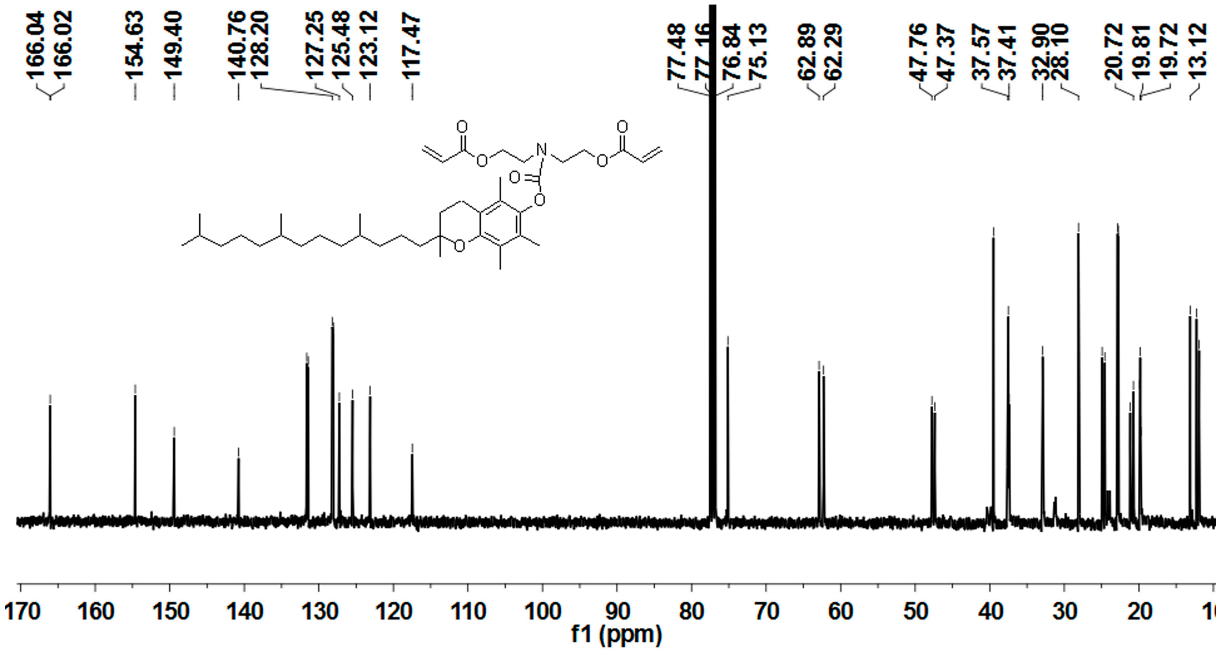

Fig. S3. ^{13}C NMR of **6a.**

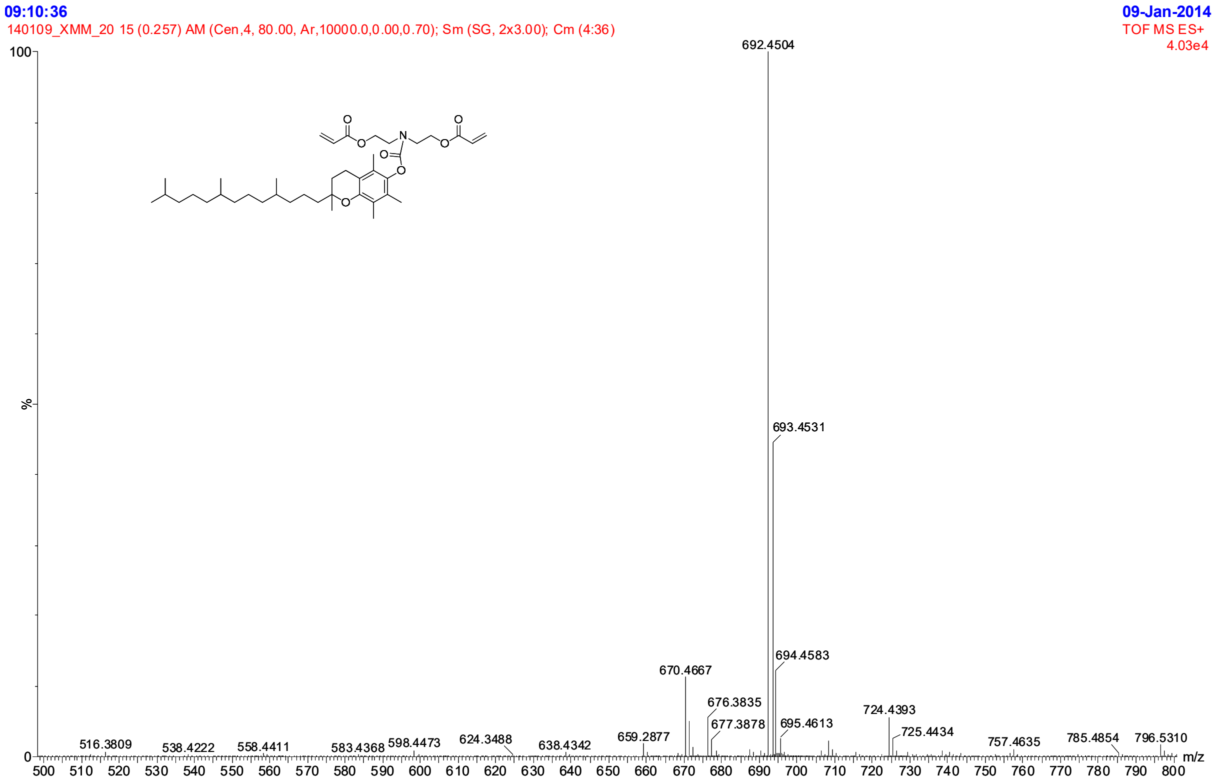

Fig. S4. HRMS (ESI) of **6a.**

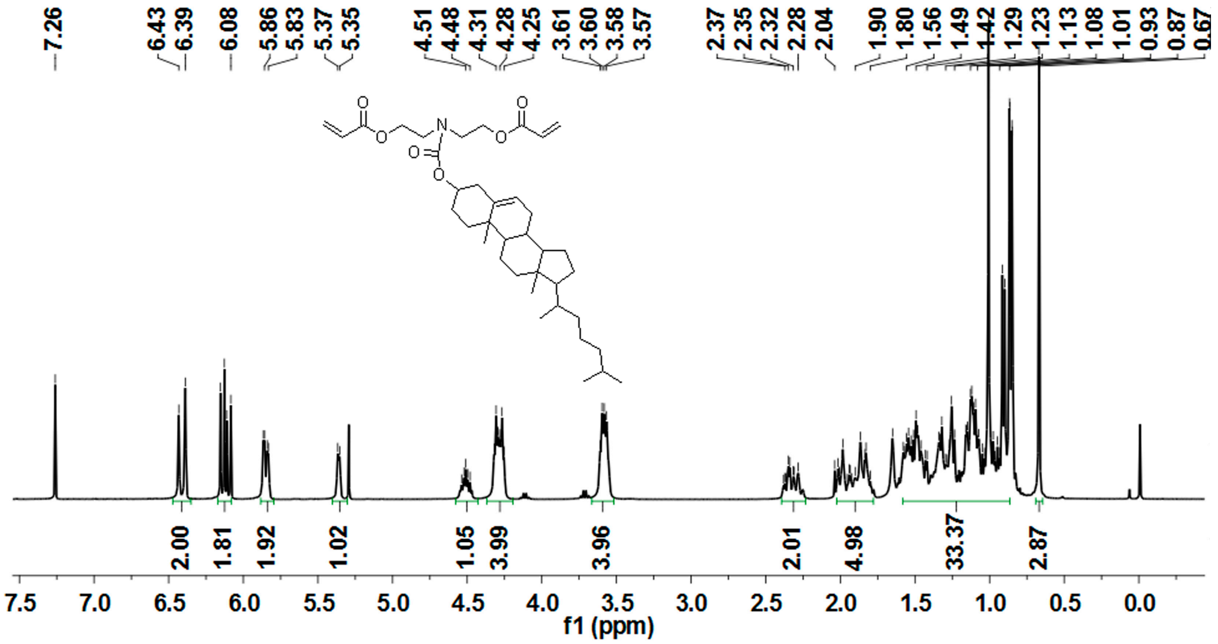

Fig. S5. ^{1}H NMR of **6b.**

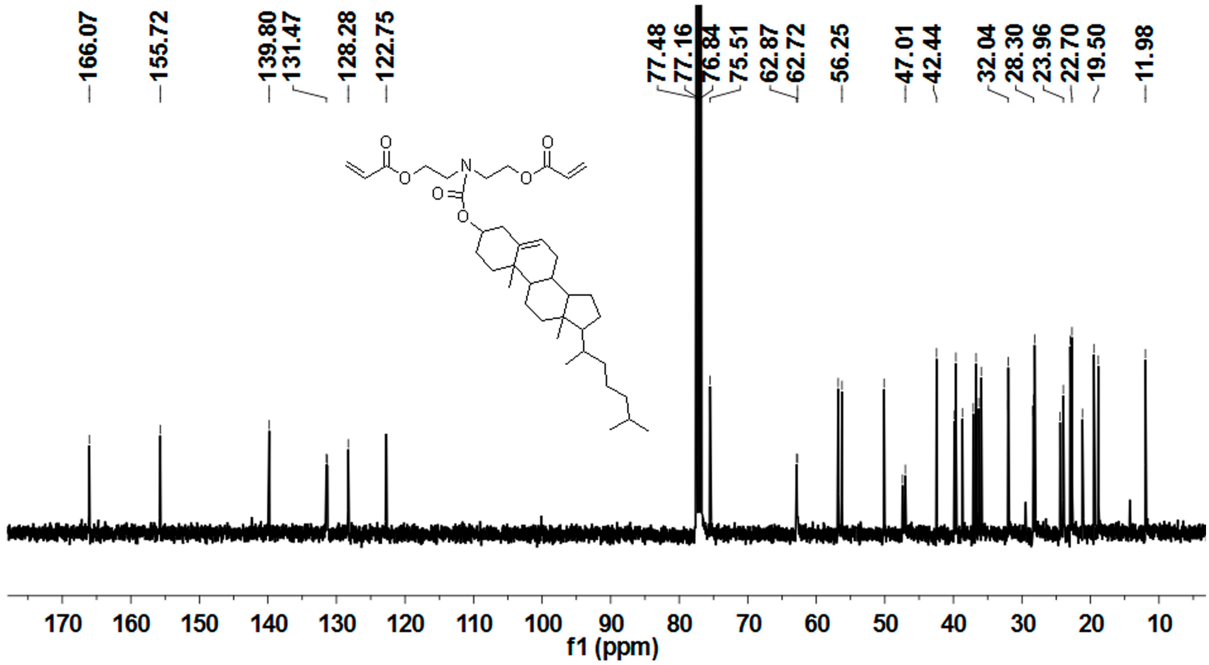

Fig. S6. ^{13}C NMR of **6b.**

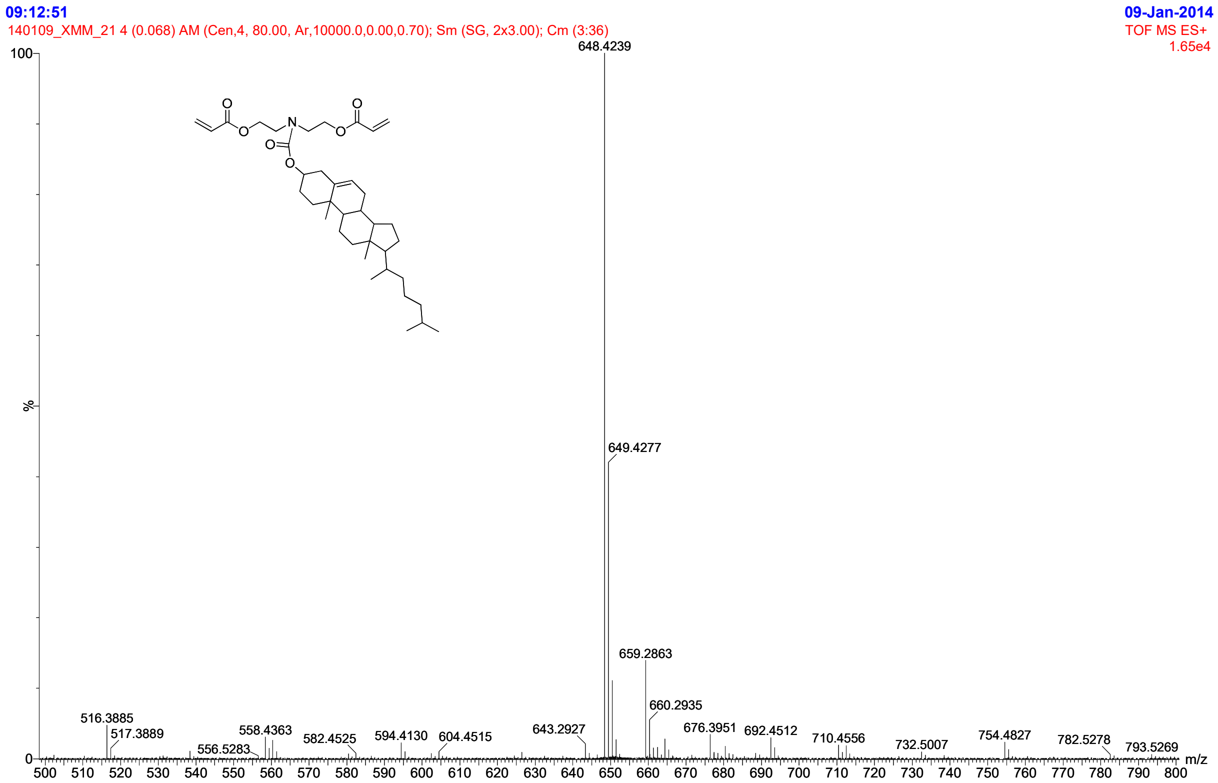

Fig. S7. HRMS (ESI) of **6b.**

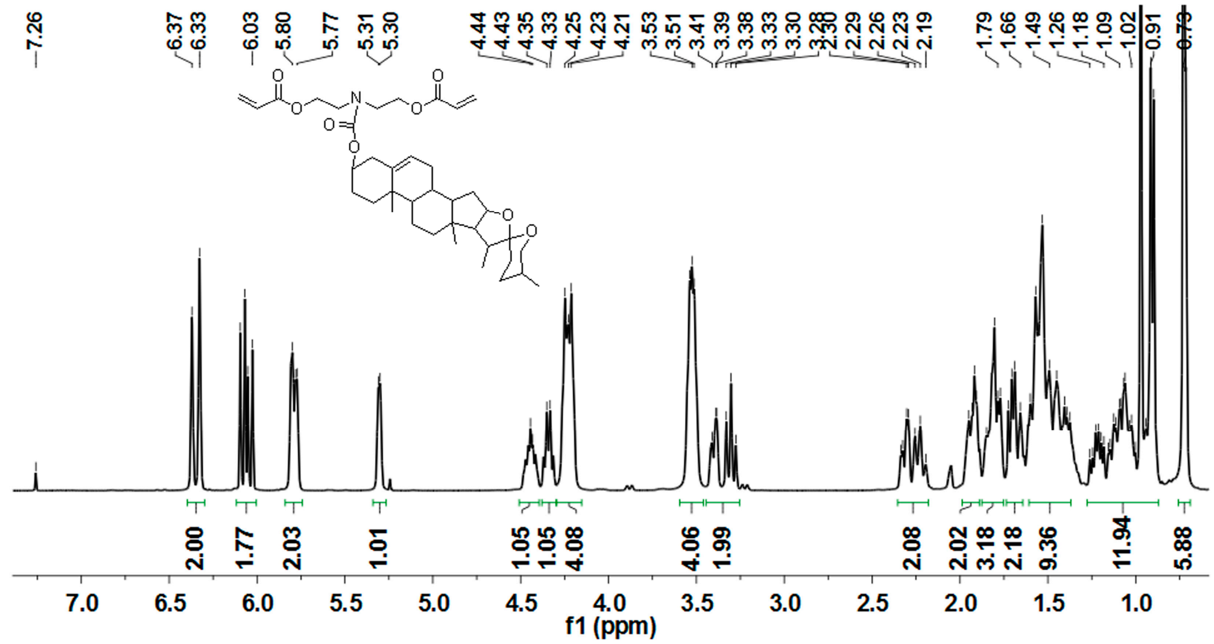

Fig. S8. ^{1}H NMR of **6c.**

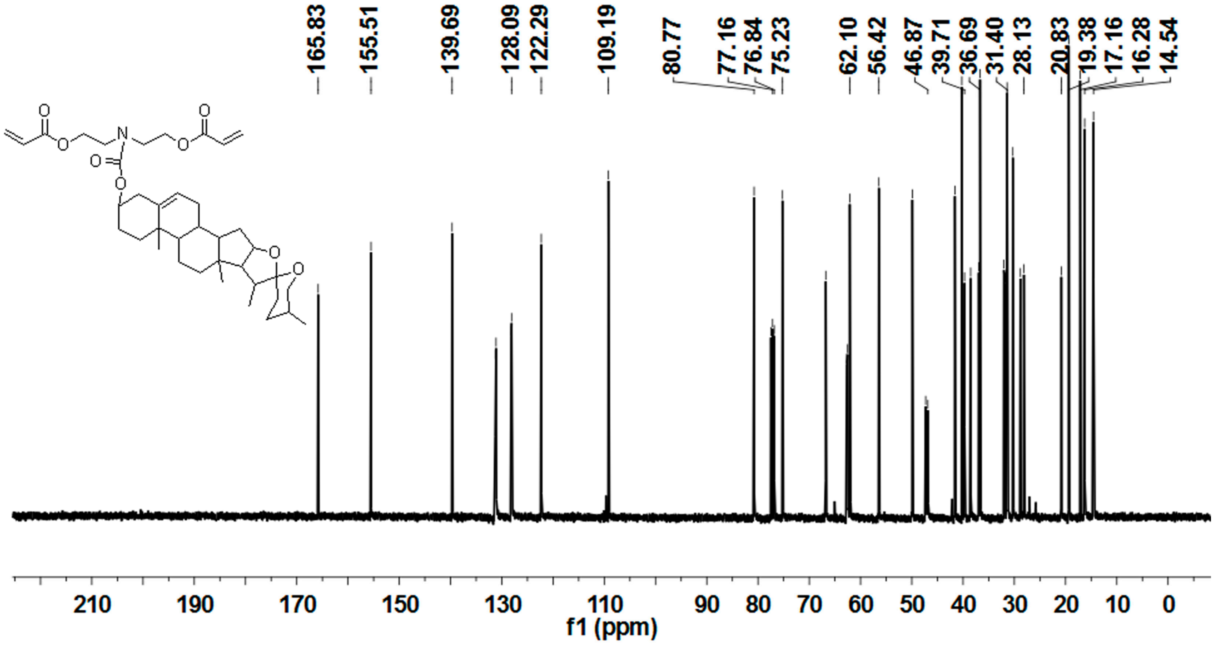

Fig. S9. ^{13}C NMR of **6c.**

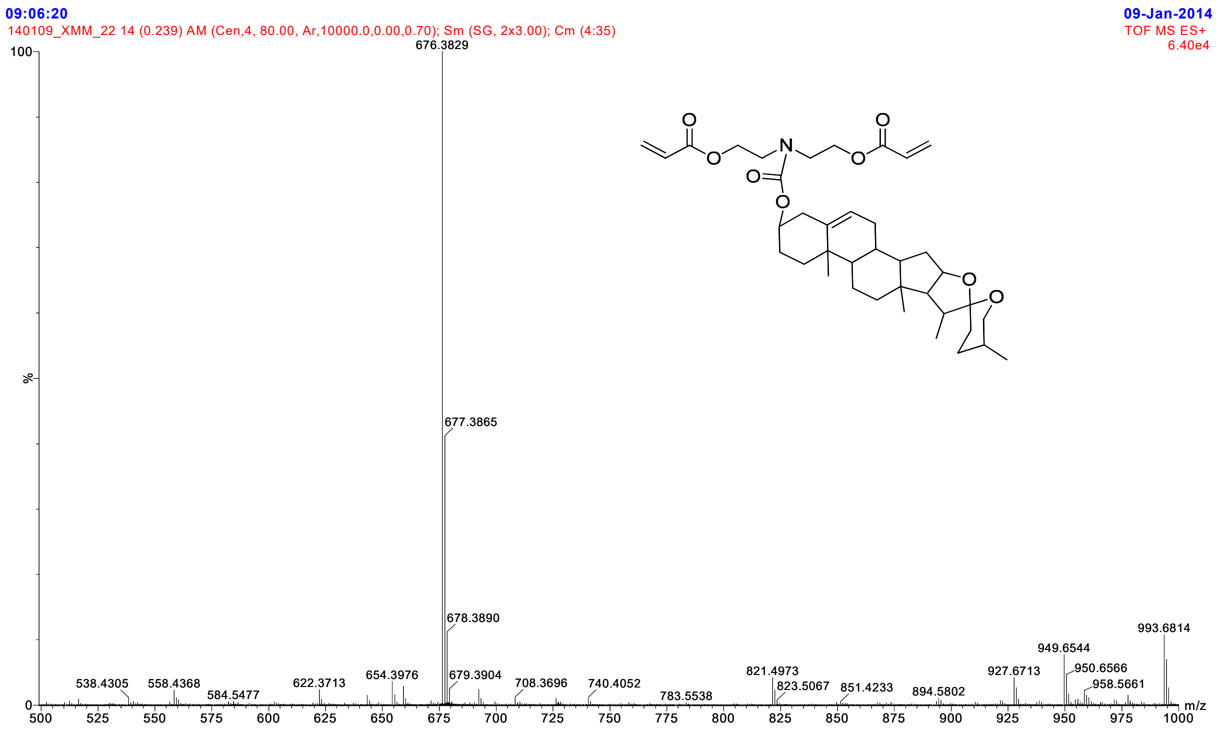

Fig. S10. HRMS (ESI) of **6c.**

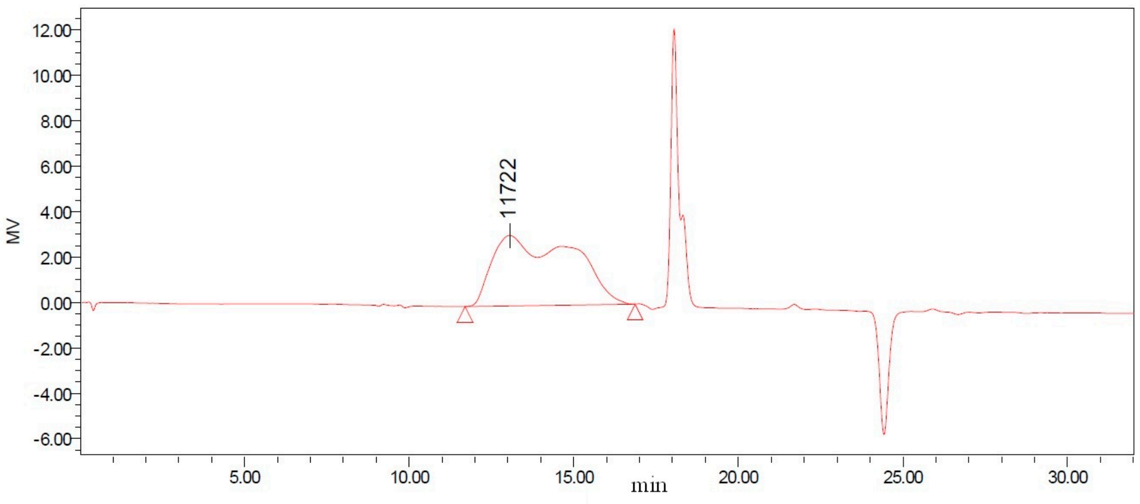

GPC result

	Distribution	Mn<br>Da	Mw<br>Da	MP<br>Da	Mz<br>Da	Mz+1<br>Da	PDI
1		4704	7880	11722	12302	16334	1.675376

Fig. S11. The GPC chromatograms of **6a.**

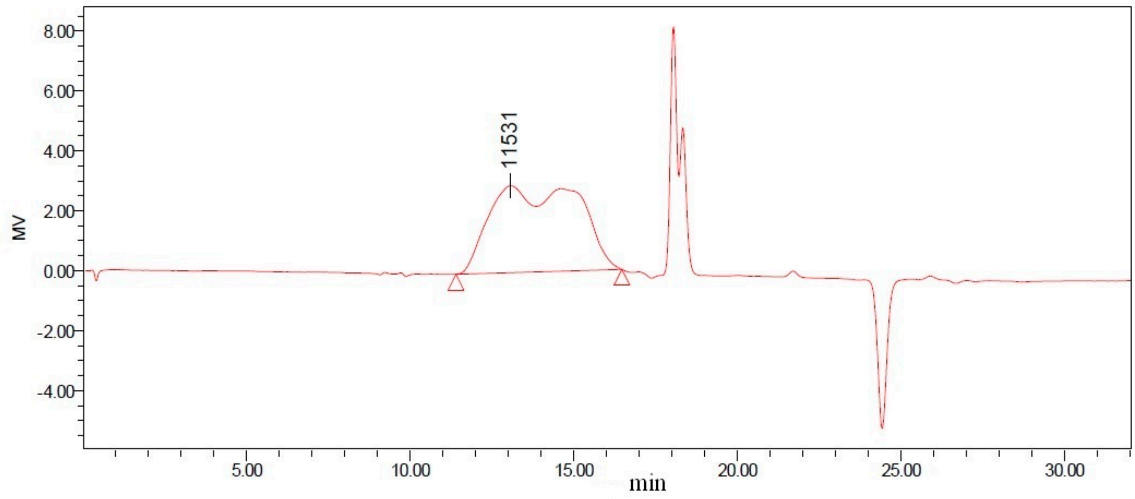

GPC result

	Distribution	Mn Da	Mw Da	MP Da	Mz Da	Mz+1 Da	PDI
1		5008	8974	11531	15701	22838	1.791719

Fig. S12. The GPC chromatograms of **6b.**

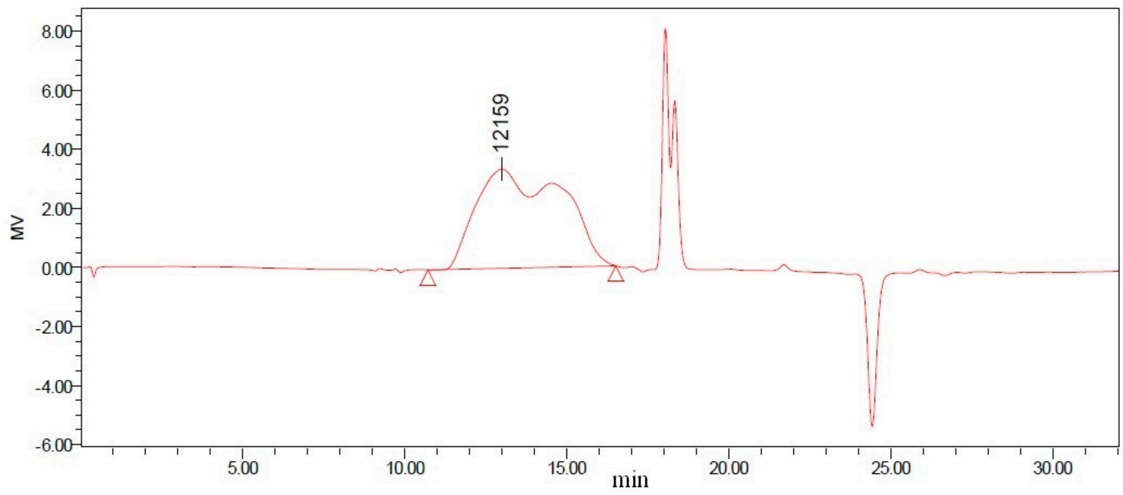

GPC result

	Distribution	Mn Da	Mw Da	MP Da	Mz Da	Mz+1 Da	PDI
1		5621	10985	12159	20476	30565	1.954375

Fig. S13. The GPC chromatograms of **6c.**
